# Imaging and micro-invasive analyses of black stains on the passepartout of Codex Atlanticus Folio 843 by Leonardo da Vinci

**DOI:** 10.1038/s41598-023-31129-2

**Published:** 2023-03-25

**Authors:** Nicolò Guarnieri, Marta Ghirardello, Sara Goidanich, Daniela Comelli, David Dellasega, Marine Cotte, Elena Fontana, Lucia Toniolo

**Affiliations:** 1 Department of Chemistry, Materials and Chemical Engineering, Politecnico di Milano, Piazza Leonardo da Vinci 32, 20133 Milan, Italy; 2 Department of Physics, Politecnico di Milano, Piazza Leonardo da Vinci 32, 20133 Milan, Italy; 3 Department of Energy, Politecnico di Milano, Piazza Leonardo da Vinci 32, 20133 Milan, Italy; 4 European Synchrotron Radiation Facility, Grenoble, France; 5 Laboratoire d’Archéologie Moléculaire et Structural (LAMS) CNRS UMR 8220, Sorbonne Université, Paris, France; 6 Veneranda Biblioteca Ambrosiana, Piazza Pio XI 2, 20123 Milan, Italy

**Keywords:** Materials science, Optics and photonics

## Abstract

This paper accounts for the diagnostic campaign aimed at understanding the phenomenon of black stains appeared on the *passepartout* close to the margins of *Folio 843* of Leonardo da Vinci’s *Codex Atlanticus*. Previous studies excluded microbiological deterioration processes. The study is based on a multi-analytical approach, including non-invasive imaging measurements of the *folio,* micro-imaging and synchrotron spectroscopy investigations of *passepartout* fragments at different magnifications and spectral ranges. Photoluminescence hyperspectral and lifetime imaging highlighted that black stains are not composed of fluorescent materials. μATR-FTIR imaging of fragments from the *passepartout* revealed the presence of a mixture of starch and PVAc glues localized only in the stained areas close to the margin of the *folio*. FE-SEM observations showed that the dark stains are localized inside cavities formed among cellulose fibers, where an accumulation of inorganic roundish particles (∅100–200 nm in diameter size), composed of Hg and S, was detected. Finally, by employing synchrotron μXRF, μXANES and HR-XRD analyses it was possible to identify these particles as metacinnabar (β-HgS). Further research is needed to assess the chemical process leading to the metacinnabar formation in the controlled conservation condition of Leonardo’s *Codex*.

## Introduction

The *Codex Atlanticus* is one of the most extensive collections of Leonardo da Vinci's drawings and writings (it collects materials from 1478 to 1519) and it is currently preserved at the Biblioteca Ambrosiana in Milan. The manuscript, after the last restoration carried out at Grottaferrata between 1962 and 1972^1^, consists of 1119 *folii* framed with a modern *passepartout*, bound in 12 volumes: each page is composed of a *passepartout* (added by restorers in Grottaferrata, with an external size about 65 × 44 cm) that frames the original fragments (*folii*) by Leonardo. The *Codex*, since 1997, is conserved in a strictly controlled and secured microclimatic environment (19 ± 1 °C and RH 55%) and stored in anti-acid cartoon directories, according to the museum standards for paper conservation^2^, while in the period from 1972 to 1997 it was conserved in Biblioteca Ambrosiana or in a bank vault without any specific environmental control. In this paper the term *folio* refers only to the original fragments produced by Leonardo Da Vinci; the term *passepartout* indicate a “sandwich structure”, composed of three modern paper layers, that has been glued to the *folio* for easy handling and display (“Structure of Grottaferrata’s passepartout and black stains optical microscopy observation”). The role of the passepartout is also that of framing the *folii* allowing Leonardo’s double-side documents to be read and examined. Finally, the term “*Folio 843*” refers to the object of cultural heritage interest (consisting of both *folio* and *passepartout* joint together) that was examined in this paper. To remind this distinction along the text, these words are in italic all over the paper.

**Figure 1:**
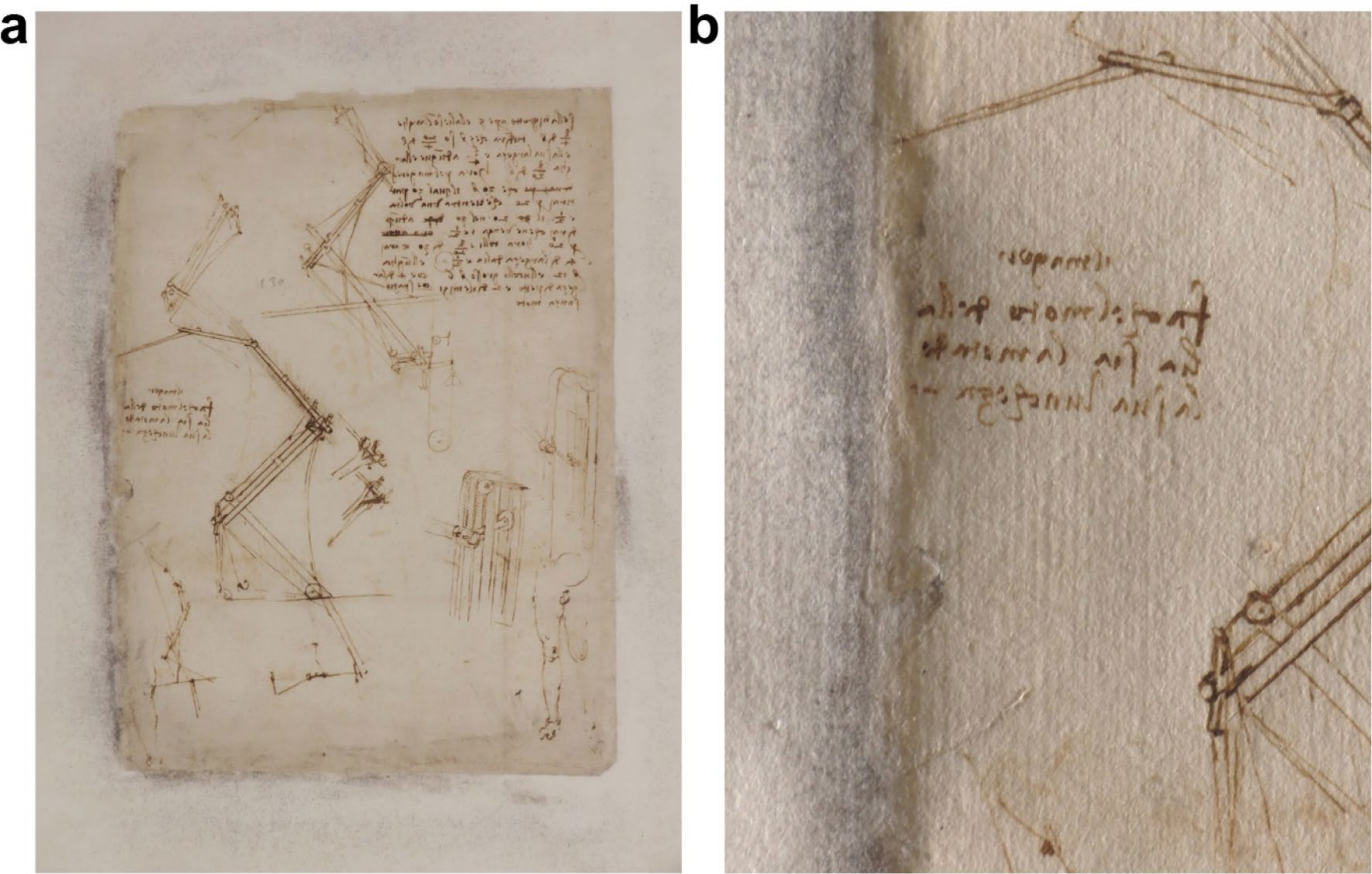
Recto of *Folio 843* (Leonardo’s *folio* size 29.6 × 22.1 cm). (**a**) Condition of *Folio 843* in May 2021. (**b**) Detail of the black stains in the central area on the left margin (images courtesy of restorer Dr. Vito Milo).

Some black-greyish stains were discovered in 2006 on the *passepartout* near the margins around the *folio*, as it is possible to observe in the case of *Folio 843* (Fig. 1). This blackening phenomenon of the *passepartout*, observed on approximately 210 pages of the *Codex* starting from *folio* 600 onward, raised great concern. Microbiological studies have been conducted since 2008, which allowed to rule out any type of microbiological attack on the *Codex* (both *passepartout* and *folio*) as the cause of the black stains^3–6^. After being sure of the safety of the *Codex* from microbiological infestation, Ambrosiana’s curators decided to face this deterioration phenomenon together with Italian Central Institute for Restoration (ICR) in 2008 to assess the conservation state of the artefact^7^. Chemical and physical investigations, including SEM–EDX and XRF analysis, allowed ICR to detect the presence of mercury in the blackened areas^7^.

The present investigation began on the occasion of the removal and replacement of the *passepartout* of *Folio 843* in 2021. The scientific study consisted of a preliminary non-invasive examination of the whole *Folio 843* by photoluminescence (PL) hyperspectral and lifetime imaging, and was then followed by laboratory analyses on *passepartout* fragments that were made available for the research*.* On these fragments we implemented a multimodal experimental protocol consisting of the micro-invasive analytical techniques typically reported in the literature to investigate historical paper degradation^8–11^, as optical and scanning electron microscopy, ATR-FTIR and Raman, and also synchrotron-based analysis (µXRF, µXANES and HR-XRD).

The goal of this multi-analytical study is to characterize the materials and the deterioration patterns of *Folio 843* and propose a hypothesis on the formation of the black stains.

## Materials and methods

### Folio 843 and its passepartout

The structure and the appearance of *Folio 843 *(Fig. 1) were subjected to a careful visual examination together with Ambrosiana’s restorer and curators. Dark stains were localized in the left margin of *passepartout* and in some areas in the central part of the right and bottom margins, approximately 1 cm around the *folio*. No clear sign of staining was detected on Leonardo’s paper. However, no more in-depth microscopic examination of Leonardo’s *folio* was allowed by curators, while it was possible to examine *Folio 843* by photoluminescence hyperspectral and lifetime imaging (“Photoluminescence hyperspectral and lifetime imaging”). The general condition of *Folio 843* was judged similar the one described by previous studies in the literature^3,5^. Later on, the restorer of Ambrosiana Library carried out the conservation intervention consisting of the detachment of Leonardo’s *folio* from the *passepartout* and the introduction of a new clean and safe *passepartout* according to recent standard preservation procedures. Grottaferrata’s *passepartout* was given available for laboratory research investigations. The size of the *passepartout* was 64.5 × 43.5 cm, with a 6 cm frame length. Fragments of *passepartout* paper were collected from the left margin where the phenomenon of blackening was more intense. Three equal strips of *passepartout* paper (from the internal to external margin of the *passepartout* frame) were cut and used respectively for: sample 1 for FESEM/EDX, sample 2 for µATR-FTIR, and sample 3 for synchrotron analyses (supplementary Fig. S1). Each strip was representative of the three differently deteriorated regions, as identified by microscopic examination (“Structure of Grottaferrata’s passepartout and black stains optical microscopy observation”). In addition, spot microscopic sampling of cellulose fibers was carried out to obtain reference µFTIR spectra of the *passepartout* paper (“Micro-Fourier transform infrared spectroscopy and microimaging”).

### Methods

Methods of investigation were selected on the basis of the following reasons: (a) to perform a rapid 1-day in-situ campaign of non-invasive investigations at the aim of examining the material differences of the *passepartout* margins and Leonardo’s *folio*; (b) to in-depth investigate chemical, mineralogical and physical nature of black stains of the *passepartout* at microscopic level*.* The applied techniques are carefully described in the following sub-paragraphs.

#### Photoluminescence hyperspectral and lifetime imaging

For photoluminescence hyperspectral imaging (PL-HSI) analysis, the *folio* was illuminated with the ultraviolet radiation produced by mercury vapor lamps (PL-S/10 UV-A, Philips) equipped with a DUG11 filter. The excitation light displays a spectrum centered at 365 nm with a full width at half maximum lower than 10 nm. The hyperspectral camera (with sensitivity in the spectral range between 400 and 900 nm and spectral resolution of about 4 nm at 600 nm) uses the Fourier Transform (FT) approach by exploiting a TWINS (Translating-Wedge-Based Identical Pulses eNcoding System) interferometer coupled to a monochromatic camera^12^. The device allows one to retrieve the hyperspectral datacube of the optical emission made of the emission spectrum at each point of the analyzed area. Photoluminescence lifetime imaging was achieved by illuminating selected areas of the *folio* through the third harmonic emission of a Nd:YAG laser (FTSS 355-50 Crylas GmbH), emitting nanosecond pulses at 355 nm. The optical emission of the *folio* was recorded by a time-gated intensified camera (C9546-03, Hamamatsu Photonics and Retiga R6, Qimaging) equipped with custom-built trigger unit and a delay generator to detect the emission in time windows of few nanoseconds at different delays with respect to pulsed excitation. Photoluminescence temporal decays detected by each pixel of the camera sensor are fitted following a mono-exponential model to reconstruct the effective lifetime map.

#### Optical microscopy

*Passepartout* paper and adhesives were observed by a Leica M205C stereo and a Leica DM6 optical microscope coupled with a MC 150 HD and Flexacam C1 camera, respectively.

#### Field-emission scanning electron microscope

Small samples (about 5 × 5 mm squares) were obtained from sample 1 and observed at high magnification by a Zeiss Supra 40 Field-Emission Scanning Electron Microscope (FE-SEM), operating in high-vacuum and equipped with the GEMINI column. The accelerating voltage was set at 3–5 kV. On the same samples, the elemental composition was determined by energy-dispersive X-ray spectroscopy (EDX) by using an Xplore-15 energy dispersive spectrometer (Oxford Instruments) up to 25 kV of accelerating voltage.

#### Micro-Fourier transform infrared spectroscopy and microimaging

Micro-attenuated Total Reflectance FTIR imaging (µATR-FTIR) was carried out using a Thermo Nicolet iN10 MX spectrometer equipped with a cooled MCT/A detector and a µATR Germanium crystal accessory. Mapping was performed on the *passepartout* fragments over a rectangular sampling area of 38 × 0.4 mm, shown in Fig. 6. In this area 1544 spectra (a grid of 4 × 386 µATR spectra) were collected with an aperture of 100 × 100 μm and a step of 100 µm. 128 scans spectra were collected at 4 cm^−1^ spectral resolution in the range 4000–750 cm^−1^. Reference µFTIR spectra of cellulose paper of the *passepartout* and starch glue were also obtained taking a micrometric sample from the verso of the *passepartout* and raw wheat starch powder, kindly provided by Ambrosiana Library’s restorer.

#### Synchrotron-based micro-X-ray analyses

Several types of synchrotron radiation-based X-ray analyses were conducted at the European Synchrotron Radiation Facility (ESRF, Grenoble, France). Synchrotron-based X-ray fluorescence (XRF) mapping and X-ray absorption near edge spectroscopy (XANES) data were acquired at beamline ID21^13^. Analyses were carried out on the three fragments showing distinct levels of alteration obtained from sample 3: almost no alteration (“*white*”), medium alteration (“*medium-dark*”) and high alteration (“*dark*”) (“Structure of Grottaferrata’s passepartout and black stains optical microscopy observation”).

Large XRF maps (approximately 4 × 14 mm) were first recorded by employing a monochromatic X-ray micro-probe (beam size defined with slit to 100 × 100 µm) with fixed energy of 4.04 keV (i.e., just at the Ca K-edge, but not strongly above, to slightly excite Ca, and excite also S and Cl through their K-edges, and heavier elements through their M-edges) and with a dwell time of 130 ms/pixel. Then, the beam was focused (beam size 0.7 × 0.45 µm, h × v), and µXRF maps were acquired on smaller regions (800 × 800 µm) of the dark sample only. The software PyMca was used to fit the XRF spectra and to separate the contribution of different elements.

Single-point fluorescence mode XANES spectra were acquired using the unfocussed beam (100 × 100 µm) by scanning the primary energy across the absorption K-edge of S (2.46–2.53 keV; energy step: 0.2 eV), of Cl (2.81–2.89 keV; energy step: 0.2 eV) and of Ca (4.03–4.13 keV; energy step: 0.25 eV). The double crystal monochromator was calibrated with a gypsum reference having the maximum absorption at the S K-edge at 2.4825 keV). 45 spectra were acquired at different points over each of the three fragments, and then averaged. Spectra were then compared to the ID21 sulfur XANES database^14^. µXANES were additionally acquired with the focused beam, at the S K-edge only, targeting specifically white and/or *dark* regions in each sample. In these conditions of high dose, the risk of beam damage was assessed by repeating several acquisitions at the same point. Results are reported in supporting information (supplementary Fig. S6). Data processing of single-point S-Kedge XANES spectra was performed with PyMCA software.

Two types of X-ray diffraction (XRD) measurements were performed, both in the framework of the “Historical Materials BAG”^15^. High angular resolution XRD (HR-XRD) measurements were collected at ID22 beamline on samples from the *dark* and *white* regions, employing a radiation energy of 35 keV. The identification of the diffraction patterns was performed using Match! and QualX software. μXRD experiments were carried out at the ID13 beamline. The μXRD branch was used to perform crystalline phase mapping using a 2.8 × 2.5 μm beam with an energy of 13 keV. Two-dimensional (2D) diffraction patterns were collected at every pixel of 2D maps and converted into 1D diffractograms by azimuthal integration, using the Jupyter Notebooks based on the PyFAI software package^16^ and analyzed with PyMCA software.

## Results 

### Structure of Grottaferrata’s passepartout and black stains optical microscopy observation

The structure of the *passepartout* applied during Grottaferrata’s restoration, was revealed through direct visual observations, during the detachment of the *Folio 843*, operated by the restorer in 2021. It is a stack of three different layers, made of Japanese paper glued together, where the top and bottom layers were superimposed for few millimeters over the *folio* and are actual sites of adhesion of the *folio* to the *passepartout*. A scheme of the layered structure is proposed in supplementary materials (supplementary Fig. S2).

**Figure 2:**
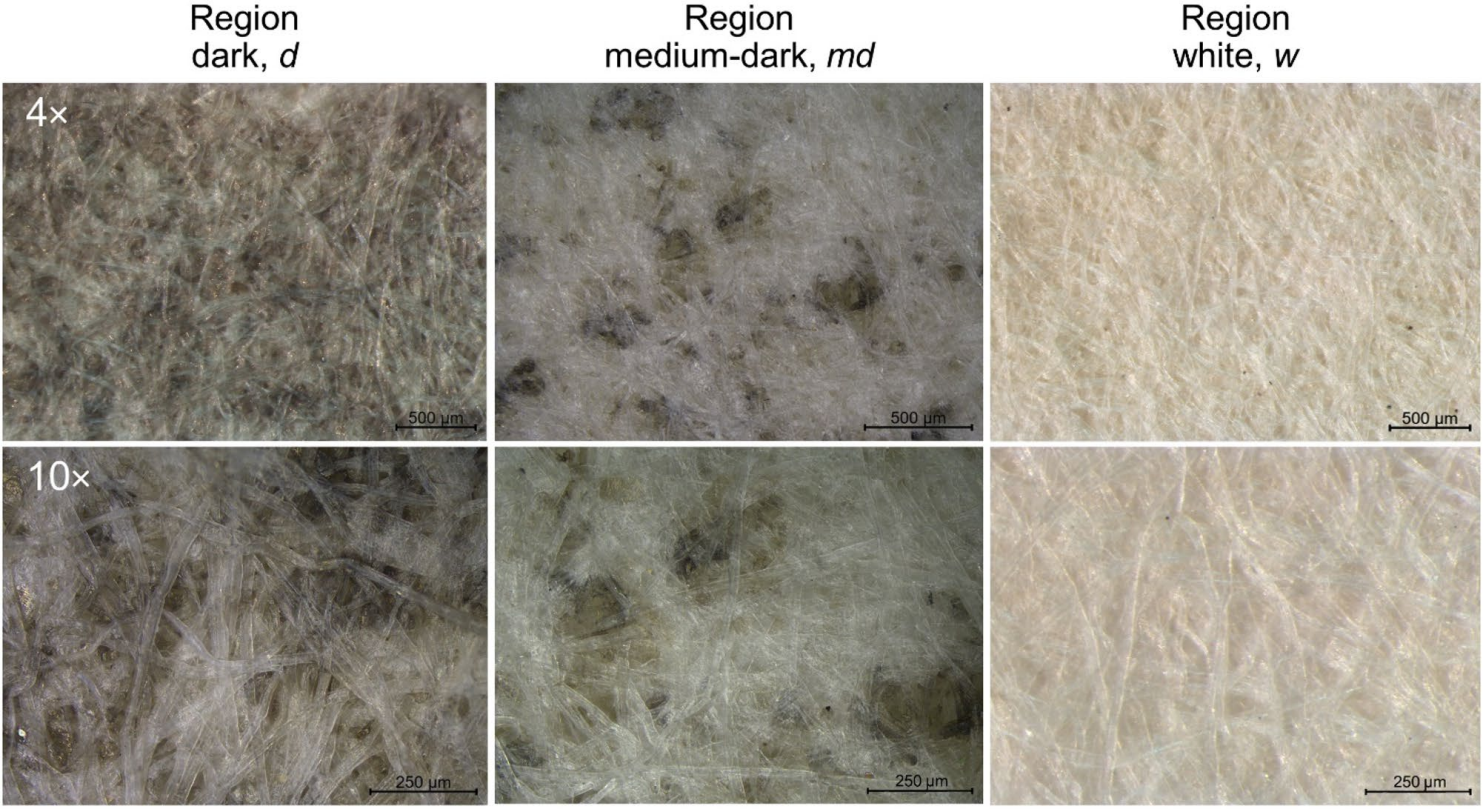
Stereomicroscopy images of the different distribution and density of the black stains in the three regions of the *passepartout*.

Since the black stain phenomenon (described in “*Folio 843 *and its *passepartout*”) shows a gradient in intensity, starting from the inner margin of the window of the *passepartout* (where the *folio* is glued) towards the external margin, the analyses were performed considering three different regions (“Folio 843 and its passepartout” and supplementary Fig. S1) at different intensities of the blackening, as assessed by stereomicroscopy (Fig. 2):

- *dark* regions (*d*), 0.5–1 cm close to the *passepartout* window margins: an intense alteration is observed; the dark stains are diffused all over the surface and the appearance of the paper is gray even at higher magnification; it is evident, at 10x, that stains are located under the surface cellulose fibers.
- *medium-dark* regions (*md*), 1–2 cm wide: the darkening phenomenon is less intense, and it is easier to distinguish the single black stain; it is even more clear that stains are located under the surface layer of cellulose fibers.
- *white* regions (*w*), 3 cm or more: no alteration of the paper is visible to the naked eye (these areas are close to the external edges of the *passepartout*). At higher magnification, no evidence of stains was observed.

### Photoluminescence hyperspectral and lifetime imaging

PL-HSI shows that the *folio* and the *passepartout* have different emission behavior, with the latter displaying a much higher intensity peaked at 420 nm (see Supplementary Fig. S3), an issue that suggests the presence of modern additives (as whiteners or brighteners). The fainter emission from the *folio* is broadly distributed from 400 to 600 nm with a main peak at 480 nm and a shoulder at 530 nm. No emission is detected in correspondence of the darkened regions.

**Figure 3:**
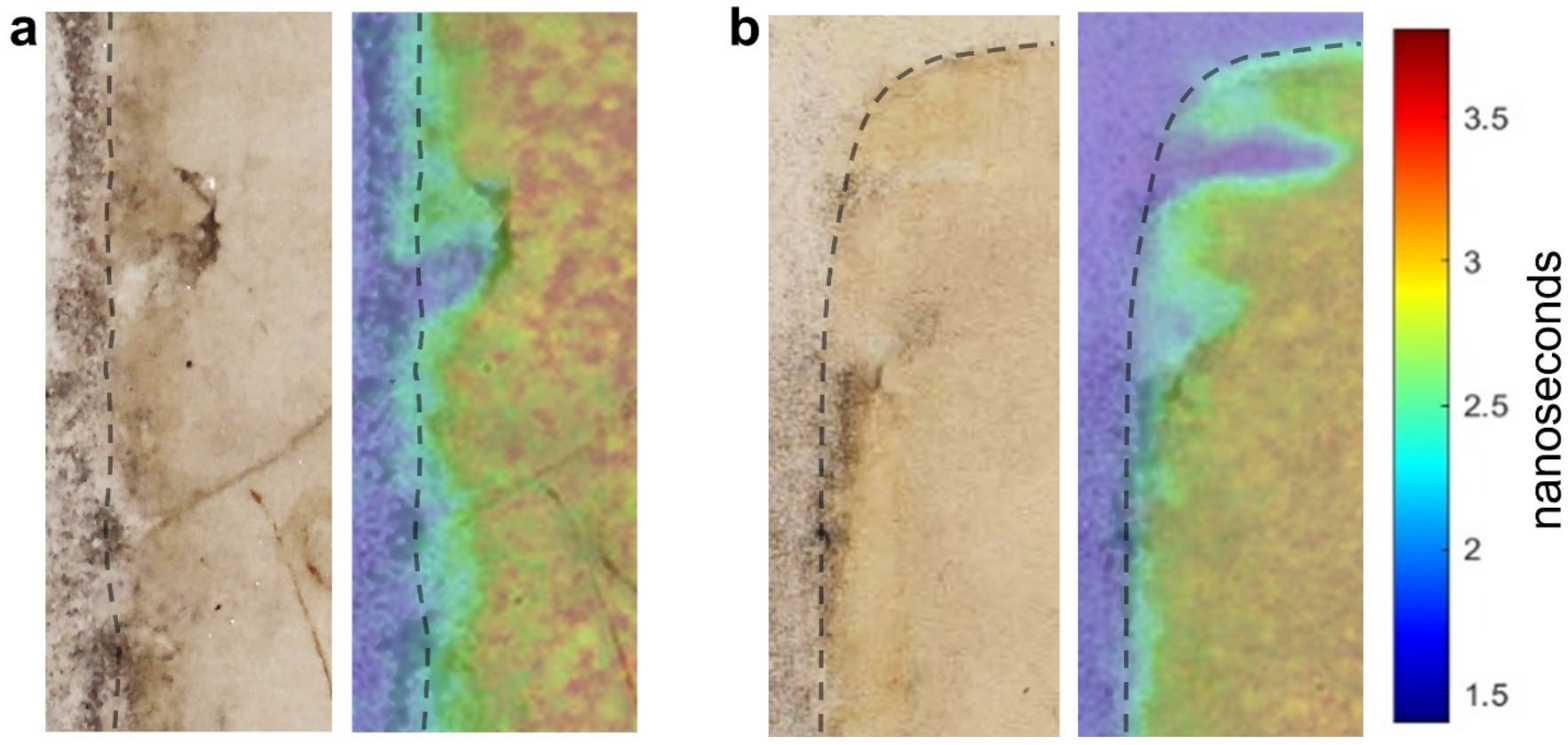
Photoluminescence lifetime imaging results of *Folio 843* performed before the detachment of the *passepartout* (**a**) central left margin and (**b**) top left angle. In both cases, both the photograph of the area (left) and the photoluminescence lifetime map (right) are proposed.

Photoluminescence lifetime analysis infers the presence of lifetime heterogeneities in correspondence with the margin between *folio* and *passepartout*. In details, in the lifetime map three different false-colored regions, corresponding to three different characteristic lifetimes, can be observed (Fig. 3): the modern *passepartout* (blue-purple color, lifetime below 2 ns), the *folio* (red–orange–yellow color, lifetime greater than 3 ns), and the margin region where the *passepartout* is superimposed to the *folio* (green color, intermediate lifetime about 2.8 ns). It is noted that some areas, which at the naked eye seem to belong to the *folio*, show instead an emission lifetime characteristic of the modern paper (identifiable by the blue-purple color in Fig. 3). This result can be explained by considering that the emission of the modern paper is much more intense than the one of the *folio.* So, it is reasonable to suppose that in these areas the *passepartout* is superimposed to the *folio* and its emission masks the one from the *folio*. Furthermore, observing the margins between ancient and modern paper (Fig. 3 and Fig. S3) it is possible to notice the presence of irregularities in lifetime map possibly related to the procedure used to glue the *folio* to the *passepartout*, since, as mentioned earlier, they were joined through the superimposition of small and thin flaps of modern paper of the *passepartout* to Leonardo’s fragment (“Structure of Grottaferrata’s passepartout and black stains optical microscopy observation” and supplementary Fig. S2).

### Laboratory analyses on microsamples

Observations under the optical microscope show that dark regions, which appear to the naked eye as dark grey or almost black, at higher magnifications (Fig. 2) appear constituted of a set of small, blackened areas isolated from each other. These dark areas are not lying on the surface of the cellulose fibers but are incorporated inside and among the fibers themselves. Indeed, the blackening phenomenon is localized only in small inter-fibers cavities formed by the intertwining of cellulose fibers. The stereo-microscopic observations of the *passepartout* in correspondence with the internal window, where the flaps were raised during the detachment of the *folio*, allowed to easily detect that the blackening phenomenon involved only the first paper layer of the *passepartout* sandwich structure, stopping at the interface between that layer and the central one.

**Figure 4:**
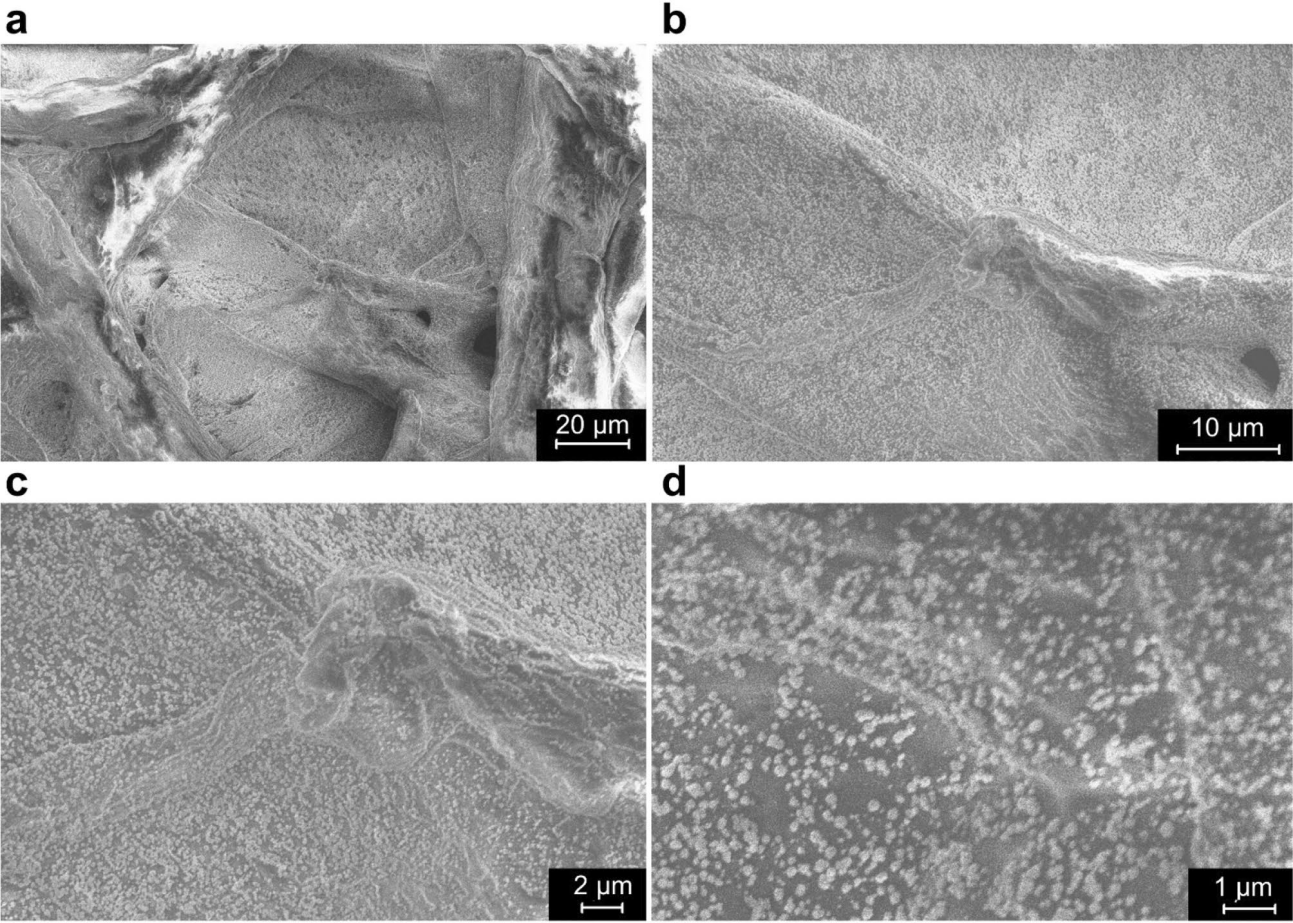
FE-SEM images of the accumulation of particles inside an inter-fiber’s cavity in the *medium-dark* region. Different magnifications are proposed: (**a**) 1.8 K×, (**b**) 5 K×, (**c**) 10 K×, (**d**) 25 K×. The size of particles varies between 200 and 300 nm. This type of structure was also found in the *dark* regions, while no detection was possible in the *white* region.

**Figure 5:**
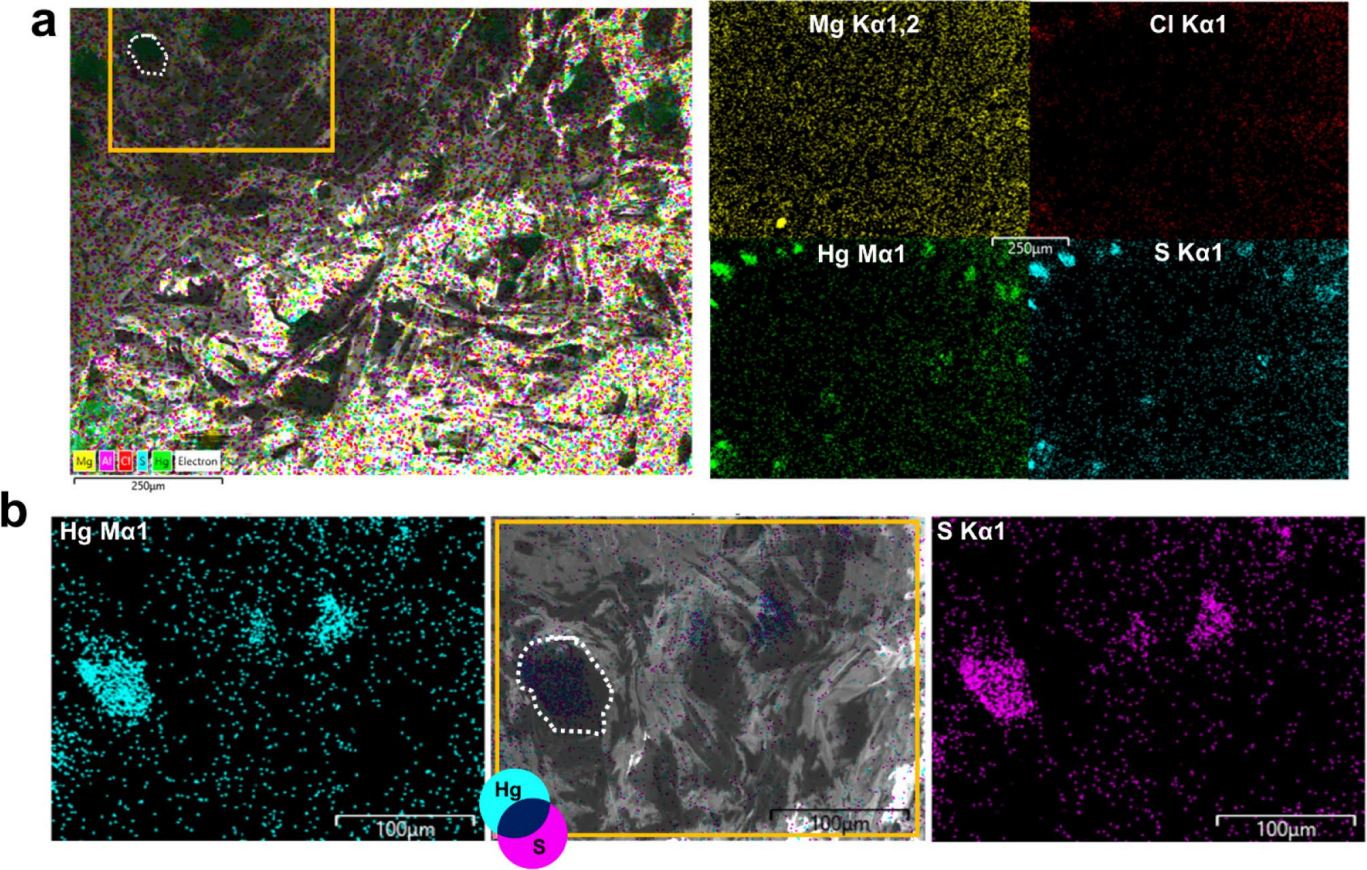
EDX elemental maps from the region *medium-dark*. (**a**) Elemental maps of Mg, Cl, Hg and S. Note that sulfur and mercury show the same spatial distribution. (**b**) Detail of a cavity and elemental map of mercury and sulfur, in this case the correspondence between the copresence of Hg and S with the shape of the cavities is well visible. The yellow rectangular shape and white dotted line highlight the same region and cavity.

FE-SEM microscopy allowed to explore the morphological features inside the inter-fiber cavities in *dark*, *medium-dark,* and *white* regions. The cellulose fibers appear clean, with flat and regular surfaces. The intertwining of the different size fibers grants the structure of the paper and its tensile strength. Exploring at increasing magnification the inter-fibers cavities in the *medium-dark* region (Fig. 4), it was possible to detect the accumulation of small, roundish particles with an average diameter of around 100–200 nm (Fig. 4d). EDX analyses collected in the *white* region show the presence of elements typical of cellulose (C, O, Na, Cl, Ca and Mg), while in the *dark* regions Hg and S are also detected (Supplementary Fig. S4). It is interesting to note that the spatial distribution of Hg and S in the *dark* and *medium-dark* regions shows a good overlapping (Fig. 5). Instead, chlorine (Cl), magnesium (Mg) and sodium (Na) are distributed homogeneously along the samples. It is found that the ratio Hg:S is 1.00:1.19. This elemental ratio and the spatial correlation between Hg and S allowed the formulation of a first hypothesis on the composition of these sub-micrometer particles located in correspondence with the black stains as made of HgS (reasonably, given the black color of stains, β-mercury sulfide).

Considering the high Raman cross-section of HgS, we tested micro-Raman spectroscopy (details of the set-up are provided in Sect. 5 of Supplementary Materials) on micrometer areas of the *folio* in correspondence with *dark* regions. However, no meaningful Raman spectrum was recorded. We suppose that this unsuccessful result is a consequence of the location of the sub-micrometer particles inside the inter-fibers’ cavities, an issue that made difficult to focus the laser excitation on them due to the strong scattering effect induced by the fibers.µFTIR measurements were performed on the three regions of the *passepartout* to study the presence of glues or other additives that could be associated with the distribution of black stains. Resulting spectra are reported in supplementary Fig. S5 and peak assignments in supplementary Table S1. Spectra of reference samples of *passepartout* cellulose paper show peaks at 3300, 2930 and 2900 cm^−1^, characteristic of the asymmetric and symmetric stretching vibrations of O–H and C–H bonds in polysaccharides, including inter and intramolecular hydrogen bond vibrations in cellulose^11,17,18^. The peak located at 1640 cm^−1^ corresponds to the vibration of water molecules absorbed by cellulose^18,19^. The absorption bands at 1429, 1370, and 1335 cm^−1^ belong to stretching and bending vibrations of –CH_2_ and –CH and –OH bonds in cellulose^19^. Peaks between 1200 and 1000 cm^−1^ belong to stretching and bending vibrations of CO bonds in cellulose and starch^20^. The peak around 900 cm^−1^ corresponds to the C–O–C ring vibration of the polysaccharide chain^17^.

The characteristic spectra of cellulose and starch present similar infrared spectra, they differ for the main peaks between 1400 and 900 cm^−1^, cellulose display four peaks at 1160, 1110, 1060, 1040 cm^−1^^17^, while starch only three at 1160, 1080, 1027 cm^−1^^20^. A further difference is the peak related to C–O–C ring vibration at 930 cm^−1^ for starch^17^ and around 900 cm^−1^ for cellulose^20^. These differences, together with the peak at 1370 cm^−1^ that is more intense in cellulose than starch, were used to identify the presence of starch glue in the p*assepartout*.

**Figure 6:**
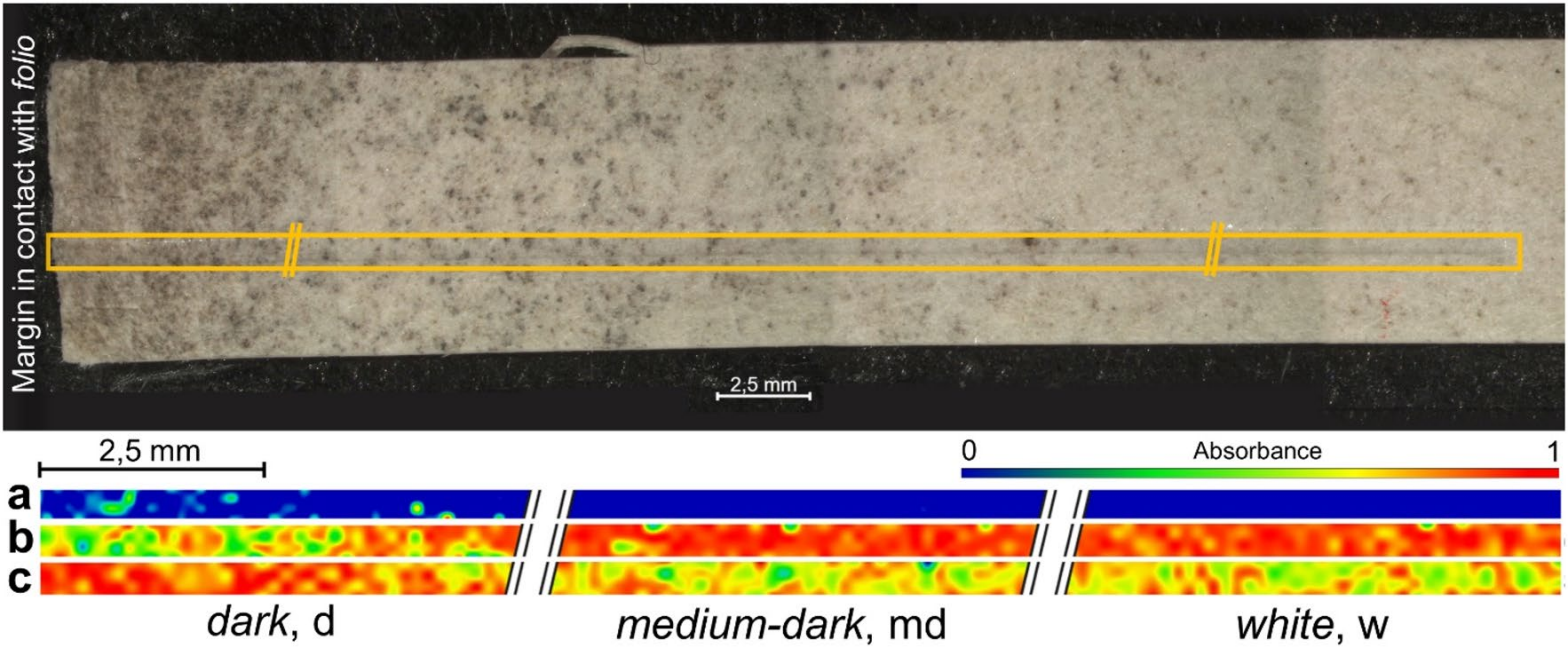
µATR imaging of *passepartout* sample 2 (“*Folio 843 *and its *passepartout*”). Top: image of the fragment where µATR imaging was performed, the total size of the fragment is around 6 × 0.7 cm; the 38 × 0.4 mm measured area is highlighted by the yellow box where the imprinting of the Ge crystal on the paper is visible; bottom: contour maps of peaks of (**a**) PVAc at 1740 cm^−1^, (**b**) cellulose at 900 cm^−1^ and (**c**) starch glue at 1027 cm^−1^. Images show the normalized absorbance in a fake color scale.

The µFTIR spectrum obtained by diamond cell in transmission mode on a microsample of the black material collected with the aid of a needle, shows two more peaks, at 1740 cm^−1^ and 1240 cm^−1^. These absorption peaks can be attributed to C=O and C–O asymmetric stretching vibrations respectively^20–22^ and it was possible to ascribe them to the presence of polyvinyl acetate (PVAc), after overlapping of a standard reference spectrum. PVAc was largely used as synthetic glue in restoration works in the second half of the last century. The comparison with reference PVAc spectrum, allowed to detect the presence of the medium-intensity peak at 1014 cm^−1^ of PVAc, that contributes to the different morphology of the absorption peaks in the region 1000–1200 cm^−1^ in the spectra of the blackened material. µATR-FTIR imaging maps were acquired on sample 2 taken from the *passepartout* (Fig. 6). The strip extends from the internal part of the *passepartout*, where the *folio* was glued (*dark* region), towards its external part (*white* region).

In Fig. 6, with the aid of a fake color map, the spatial distribution of main components is reported. PVAc (Fig. 6a, contour map of the main peak of PVAc carbonyl as. stretching at 1740 cm^−1^) has been detected only in *dark* regions, 5 mm close to the margin, where the discoloration phenomenon is higher. Despite the presence of black stains also in the *medium-dark* region, the contour map does not show the presence of PVAc in the remaining part of the analyzed fragment. The contour map of cellulose (Fig. 6b) was obtained by mapping the cellulose C–O–C ring vibration at 900 cm^−1^, while starch glue presence was evaluated with the contour map of the C-O stretching vibration at 1027 cm^−1^ (Fig. 6c). These maps show that starch glue is concentrated mainly in the *d* region (Fig. 6c, red color in *d* region), while in *md* and *w* regions starch shows a homogenous medium intensity (green-yellow color). Summarizing the infrared spectroscopic results, it can be affirmed that a mixture starch with minor addition of PVAc glue has been detected in the first 5 mm of *dark* region of the *passepartout*, close to the internal margin.

### Synchrotron analyses on microsamples

**Figure 7:**
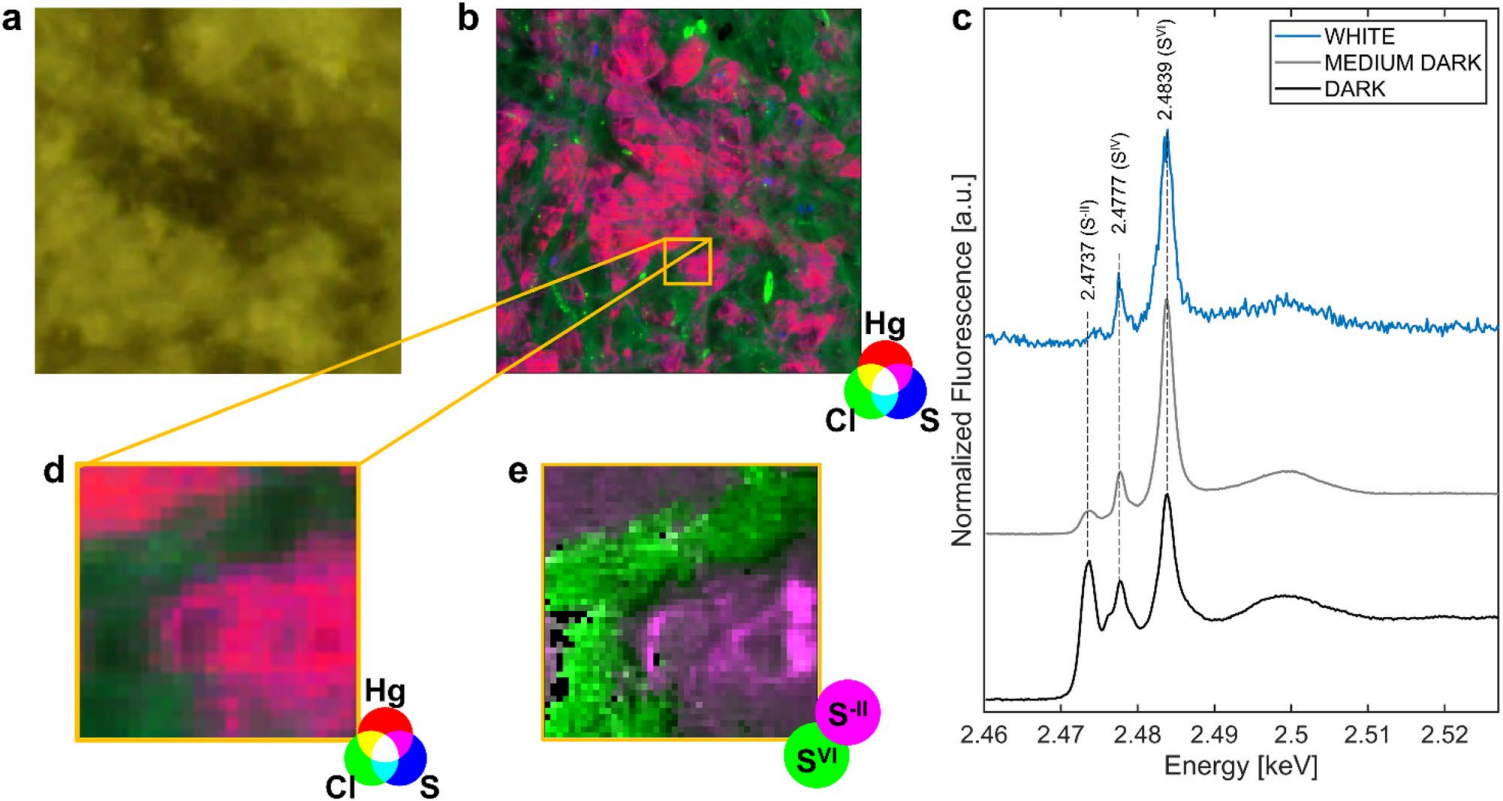
(**a**) Visible light image of the black stains region analyzed from the *dark* region. (**b**) RGB composite of SR µXRF maps of Hg (red), Cl (green) and S (blue) [step size (h × v), 4 × 4 μm; map size (h × v), 800 × 800 μm; exp. time, 100 ms/pixel; energy: 2.825 keV]. The magenta color indicates the region where mercury and sulfur signals overlap, following the shape of the black stain. (**c**) Macro-XANES spectra recorded at S K-edge (average of 45 spectra) of *white*, *medium-dark* and *dark* regions. Peak at 2.4737 keV corresponds to the sulfides (S^-II^), peak at 2.4777 keV to the sulfites (S^IV^) and peak at 2.4839 keV to the sulfates (S^VI^). It is worth noting that the intensity of the *white* region (blue line), before normalization, is almost zero, meaning that S is less concentrated in the white sample. (**d**) Detail of RGB composite of SR µXRF maps of Hg (red), Cl (green) and S (blue). (**e**) Selected AOI for hyperspectral 2D µXRF maps at S K-edge. The RGB composite image obtained by selecting ROIs in the hyperspectral dataset shows the distribution of sulfides (S^-II^) and sulfates (S^VI^) species.

Macro and µXRF maps of the three regions (*d, md, w*) confirmed the results obtained by EDX analyses, inferring the presence of Hg, S, Ca, Al, Cl, Si, K (supplementary Fig. S7). In particular, Hg was detected mainly in the *d* region, in a smaller amount in the *md*, while it was not detected in the *w* region. A similar distribution was found for sulfur, although it was also detected, in low amount, in the *white* regions. Ca, Al, Cl, Si and K were homogeneously distributed in all the three regions. µXRF map performed on a small portion of the *d* region in correspondence with the black stain (Fig. 7a) shows at the microscale a co-localization of Hg and S, which follows almost perfectly the shape of the dark stain. The magenta color in XRF map of Fig. 7b,d (which indicates the overlapping presence of Hg and S) also extends into areas that do not appear black in visible image. This can be explained by the X-ray penetration that allows to detect Hg and S below the surface of cellulose fibers. The areas where the co-localization was detected (magenta color) were constituted mainly by sulfides, as shown in Fig. 7e.

Macro-XANES spectra at Cl and Ca K-edges did not highlight any difference between the three samples (Supplementary Fig. S8), showing that these two elements are not involved in the blackening process. The Ca K-edge XANES spectra resemble that of amorphous calcium carbonate^23^, while the the Cl K-edge spectra do not match any of Hg-Cl compounds^24,25^. Its attribution remains uncertain, but we can exclude Hg-Cl bonds. Conversely, spectra acquired at S K-edge (Fig. 7c) exhibited remarkable difference between the three regions, both in signal intensity (high in *dark* and *medium-dark*, low in *white* region) and sulfur oxidation states (mainly sulfates (S^VI^) and sulfites (S^IV^) in *white*, also sulfides (S^-II^) in the *dark* and *medium-dark*). µXANES spectra were also acquired with a micro-probe, specifically in *dark* and in *white* regions. It confirms that in *dark* regions, sulfides are the main sulfur species, while sulfates are dominant in the *white* regions (Supplementary materials Fig. S9). This suggests a predominant formation of sulfides associated with the blackening. In few black points, the resolution was sufficiently good to collect signal on sulfides only, and the spectra show a good agreement with the metacinnabar β-HgS reference (Fig. S9), strengthening the hypothesis of this compound being the cause of the black stains.

**Figure 8:**
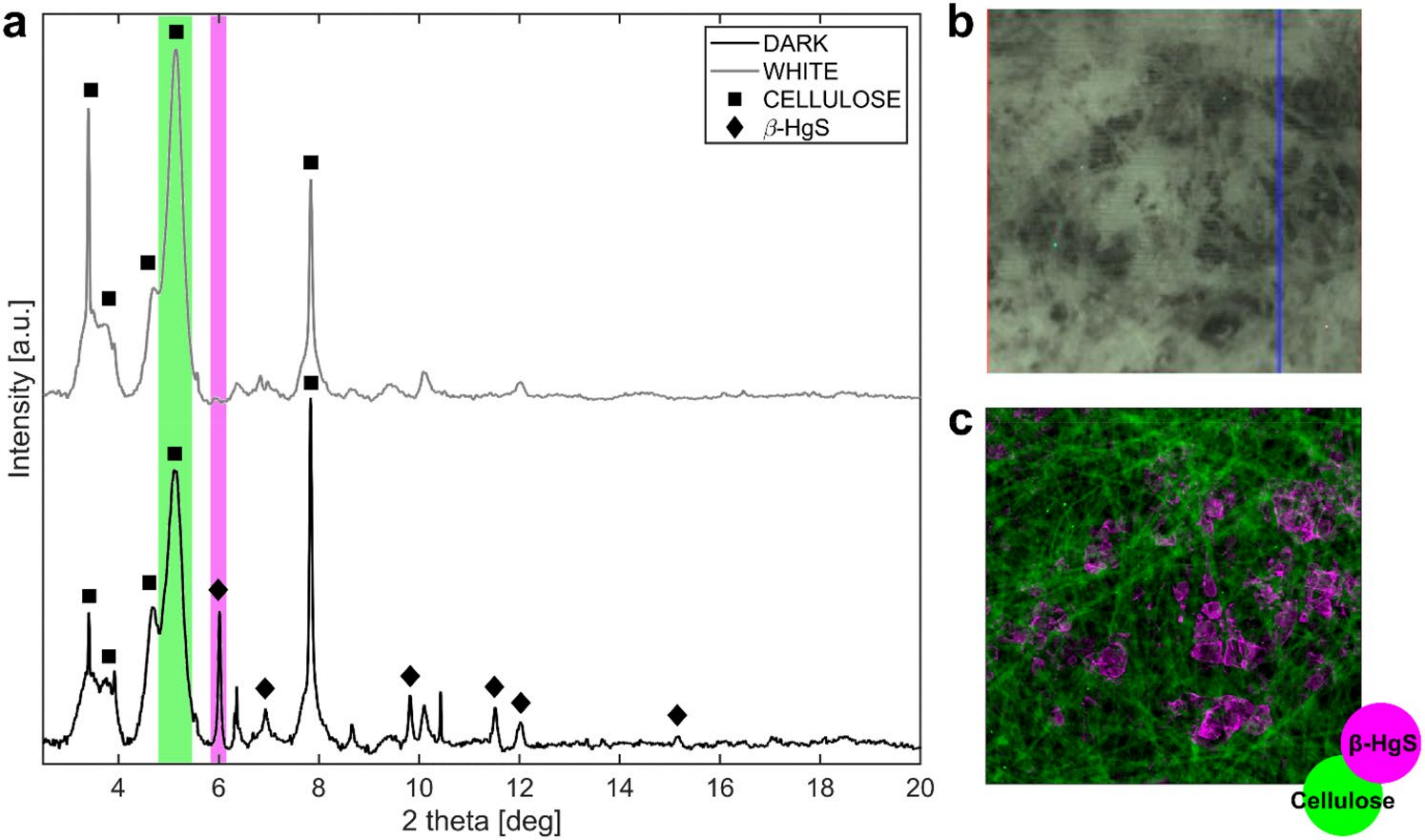
(**a**) HR-XRD spectra patterns of the *white* (grey) and *dark* (black) regions (radiation wavelength 0.35430407 Å). Squares and diamonds highlight the peaks related to cellulose and β-HgS respectively. (**b**) Visible light photograph of the area of the dark sample where SR µXRD mapping was performed. (**c**) SR µXRD images calculated by integrating XRD intensity over ROIs of XRD pattern, shown as green and purple rectangles in (**a**) for cellulose and metacinnabar respectively.

The* d* and* w* samples from the *passepartout* were finally analyzed with synchrotron HR-XRD and µXRD (Fig. 8). Main peaks of cellulose (square symbol in Fig. 8) were detected in the two samples. Other peaks can be related to the presence of metacinnabar (β-HgS, diamond symbol in Fig. 8), definitively confirming the hypothesis made from XANES data. The spatial distribution of Hg (obtained by µXRF mapping) corresponds to the blackened areas of the *folio* and to the spatial distribution of β-HgS (identified by µXRD mapping), allowing to associate blackening to the presence of this compound.

## Discussion

The combination of various non-invasive and micro-analytical techniques allowed shedding a completely new light on the blackening of the *passepartout* of Leonardo’s Codex Atlanticus. Notably, µFTIR infers the use of PVAc, employed in an aqueous mixture with starch glues. Interestingly, this adhesive mixture was applied only in some regions of the *folio* to join and strengthen its adhesion with the *passepartout*, probably applying it by brush near the internal margin, where the *folio* was joint to the multi-layer *passepartout* window. The detection of this gluing mixture has been considered critical by Ambrosiana’s restorer. Actually, the presence of this synthetic polymeric glue could be regarded as a source of a weak acidic environment in the cellulose matrix of the paper, according to the deterioration mechanism assessed also in mild museum conditions^26^.

The glue was probably applied around the internal margin and near the *folio*, before sealing the sandwich structure with the upper layer of the *passepartout*. Then the structure was placed under the press to ensure its excellent gluing and flatness. This is probably the reason why the dark stains do not appear on the surface of the fibers, but as incorporated in the cellulose matrix below some fibers. This hypothesis also explains why the blackening phenomenon was visible only on the recto of the *passepartout*, that is between the top and the central paper layers of the sandwich structure of *passepartout*.

More interestingly, in the *dark* regions of the *passepartout* we detected the presence of sub-micrometer roundish particles located among the paper fibers and identified as β-HgS. This last evidence leads to the final hypothesis that the glue mixture originally contained a mercury-based salt together with PVAc and starch. Indeed, while mercury is present along the margins as β-HgS, as undoubtedly evidenced by synchrotron µXRD, it is unreasonable that this compound could have been intentionally added to the adhesive mixture of the *passepartout*, since β-HgS crystals have a characteristic black color, which would have altered the white color of the *passepartout* and contaminated the *folio*. Instead, it is possible that, originally, the Hg source could be a white mercury salt added to the glue as anti-vegetative compound to prevent microbiological attacks.

From the few available papers in the literature on this subject, and from the memory of some paper restorer, mercuric dichloride (HgCl_2_) is the main mercury salt employed for its anti-vegetative action^27^. It is a white powder moderately soluble in water, and hence also in a water-based gluing mixture. Therefore, the presence of mercury may be attributed to this salt dissolved in the adhesive mixture.

Further studies are needed to explain the source of sulfur. Two hypotheses can be considered: from gaseous atmospheric SO_2_ resulting from industrial processes^28^ that was a quite relevant pollutant in Milan’s atmosphere till the end of the twentieth century, or from residual sulfur compounds present in the adhesives, deriving from their production or from whitening additives. Some studies^29–31^ report the formation of HgS in the β phase following the exposure of mercury salts to H_2_S or Na_2_S.

The formation reaction of β-HgS particles cannot be easily explained in the conservation conditions of the *Codex*. From the literature, different studies report the formation of HgS nanoparticles starting from mercury nitrate^32,33^, acetate^34,35^ or chlorides^36,37^ precursor in aqueous solution with thio-organic molecules. Also, HgCl_2_ biotransformation in aerated algal cultures has been investigated^38^. The conditions considered in these studies are quite distant from the conservation conditions of the *Codex*.

Referring to literature studies dealing with the blackening of red cinnabar pigment (α-HgS) in painting artworks^24,39–41^, the papers show that the phenomenon is promoted by the exposure to chlorine ions, light and humidity. The formation of mercury-sulfur-chlorine compounds is assessed, such as kenhsuite (γ-Hg_3_S_2_Cl_2_)—rare mineral identified for the first time^24^—together with corderoite (α-Hg_3_S_2_Cl_2_) and Calomel (Hg_2_Cl_2_). Corderoite, being a mineral of purplish grey color, has been identified by some authors^24^ as the compound that might be held responsible for the discoloration of cinnabar. In these studies, β-HgS was not detected in any of the altered samples; however, some authors^39^ affirm it could be present in the form of small particles, perhaps of nanometric dimensions and possibly not well crystallized, and therefore difficult to detect. They also report that the black color of degraded cinnabar can be explained by the presence of metallic mercury (Hg^0^)^24,39,41^ and a mechanism of formation is proposed^40^ supported by electrochemical experiments.

In the present study, evidence of the spontaneous formation of nanostructured β-HgS has been pointed out in controlled environmental conditions, i.e., absence of light and low humidity regime. Starting from the hypothesis of the presence of HgCl_2_ in the gluing mixture near the margin of the *folio*, one can consider that mercury-sulfur-chlorine intermediate compounds might play a role also in the chemical atmospheric process towards metacinnabar. In any case, it is worth clarifying that these compounds have not been detected in the present investigation. Further studies are required to propose a robust mechanism of the formation of β-HgS and to verify if the exposure of HgCl_2_ to different sulfur sources, in mild environmental conditions for paper artwork preservation, could lead to the metacinnabar black phase.

## Conclusions 

The aim of this work was to study the blackening phenomenon (black stains) that appeared on the *passepartout* of *Folio 843* of Leonardo da Vinci’s *Codex Atlanticus*, conserved at Ambrosiana Library in Milan. Following the detachment of the *Folio 843* from the *passepartout,* it was discovered how the two were integrated together during Grottaferrata restoration, giving rise to a sandwich-like structure. Preliminary photoluminescence imaging studies highlighted that black stains are not composed of any fluorescent material and further allowed to visualize areas where the modern*passepartout*is superimposed to the original *folio* . The presence of the blackening phenomenon has been attributed to the formation of β-HgS sub-microparticles inside the inter-fibers’ cavities of the cellulose of the *passepartout*. A mixture of PVAc and starch glues was detected in the area closer to the *folio*, which is the area that appears darkened. It remains to be determined whether degradation reactions of this synthetic glue (together with paper hydrolysis) might have played a role in promoting the blackening phenomenon.

The presence of mercury could be associated with the addition of an anti-vegetative Hg salt (probably HgCl_2_) inside the glue mixture (starch and PVAc). The mixture may have been applied only in some regions near the *folio* to ensure adhesion and to prevent microbiological attack to the Codex. Further research steps are needed to assess the chemical process that led to the formation of metacinnabar (β-HgS) in the conservation condition of the *Codex Atlanticus*: it has been hypothesized the arrival of sulfur from the environment as a pollutant (SO_2_) or from additives used in the glue, which could lead to the reaction with mercury salts and the formation of black metacinnabar particles, responsible for the black stains.

## Supplementary Information

Supplementary Information.

## Data Availability

The data used for this study can be obtained from the corresponding author on reasonable request.
