# Supplementary material for: Imaging and micro-invasive analyses of black stains on the passepartout of Codex Atlanticus Folio 843 by Leonardo da Vinci
Source: Sci Rep. 2023 Mar 25;13:4902. doi: 10.1038/s41598-023-31129-2 (PMC10039911; doi:10.1038/s41598-023-31129-2)
Supplement: Supplementary file 1 — Supplementary Information. [file 41598_2023_31129_MOESM1_ESM.docx]

**Imaging and micro-invasive analyses of black stains on the *passepartout* of *Codex Atlanticus* *Folio 843* by Leonardo da Vinci**

Nicolò Guarnieri^1^, Marta Ghirardello^2^, Sara Goidanich^1^, Daniela Comelli^2^, David Dellasega^3^, Marine Cotte^4,5^, Elena Fontana^6^, Lucia Toniolo^1^*

^1^ Politecnico di Milano, Dept. Chemistry, materials and chemical engineering, Piazza Leonardo da Vinci 32, 20133, Milan, Italy

^2^ Politecnico di Milano, Dept. of Physics, Piazza Leonardo da Vinci 32, 20133, Milan, Italy

^3^ Politecnico di Milano, Dept. of Energy, Piazza Leonardo da Vinci 32, 20133, Milan, Italy

^4^ European Synchrotron Radiation Facility, Grenoble, France

^5^ Sorbonne Université, Laboratoire d’Archéologie Moléculaire et Structural (LAMS) CNRS UMR 8220, Paris, France

^6^ Veneranda Biblioteca Ambrosiana, Piazza Pio XI 2, 20123, Milano, Italy

*lucia.toniolo@polimi.it

Supplementary materials

# Passepartout sampling


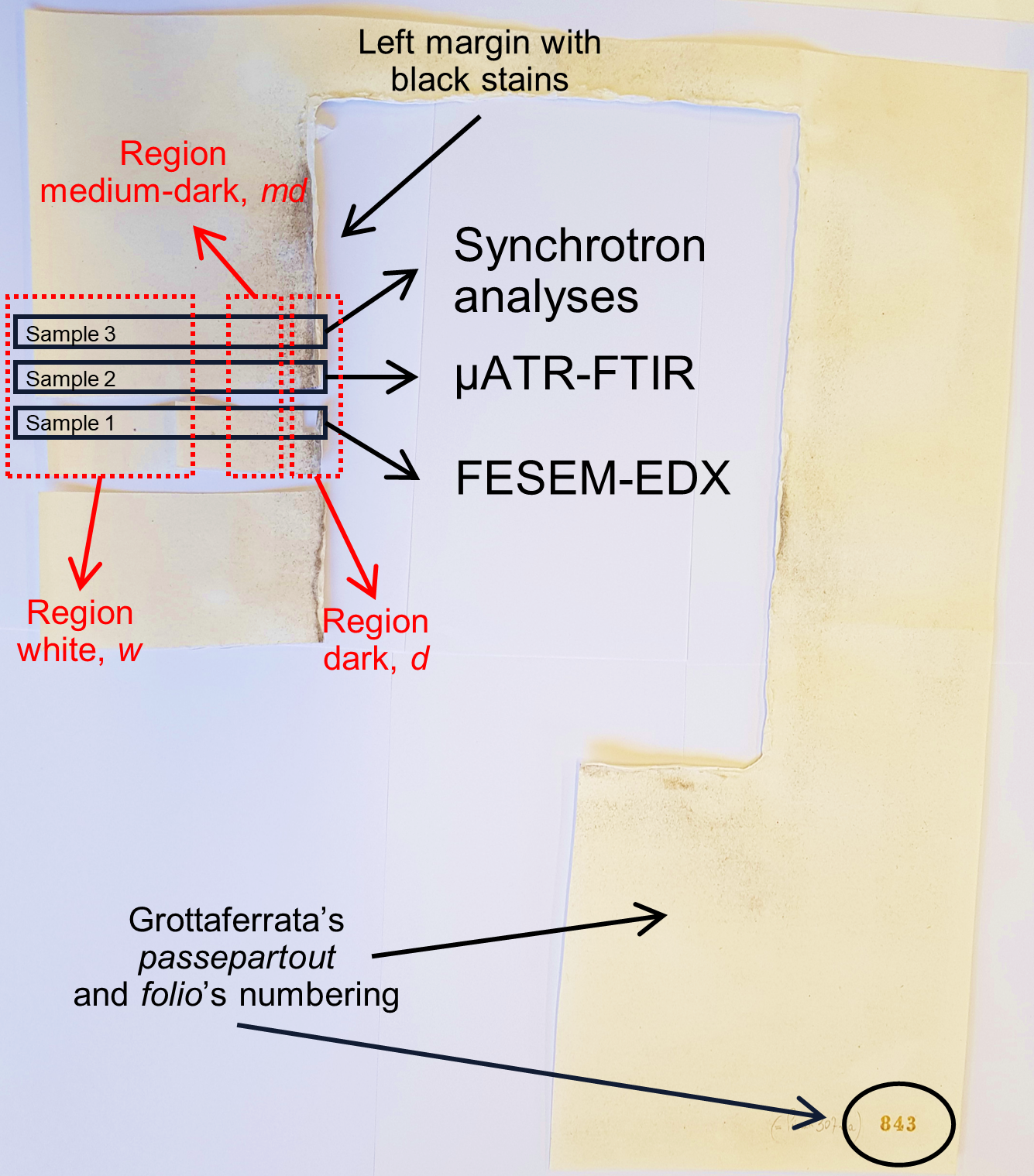


**Supplementary Fig. S1.** *Folio 843*. Part of Grottaferrata’s *passepartout* made available for laboratory analyses. Three equivalent strips of paper (about 6.0×1.0 cm) were cut from black stained left margin of the *passepartout*. Each of them showed the three examined regions: dark, medium-dark and white. Sample 1 was analysed by FESEM-EDX; sample 2 by µATR-FTIR and sample 3 by synchrotron-base techniques.

# Structure of the passepartout


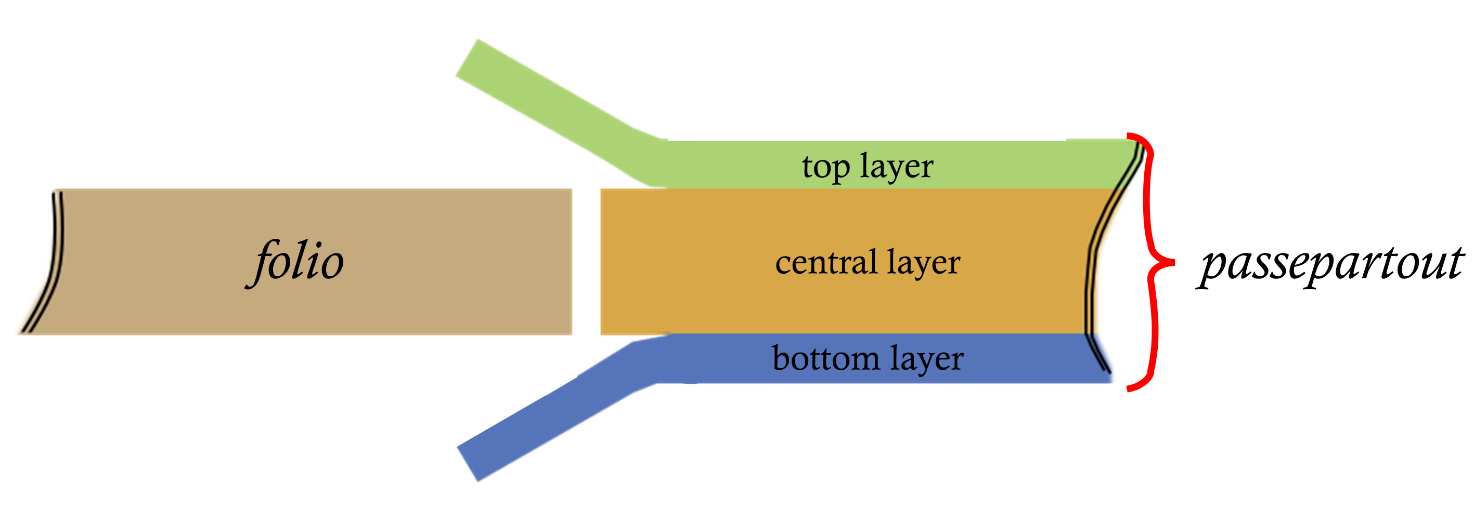


**Supplementary Fig. S2.** Cross-section of the *Folio 843* at the interface between the *folio* and the *passepartout*, the *passepartout* is composed by three layers: the top and bottom layers are glued to the *folio*.

# PL-HSI analysis

PL-HSI shows that the *folio* and the *passepartout* have different emission behaviour, with the latter displaying a much higher intensity peaked at 420 nm (Supplementary Fig. S3), an issue that suggests the presence of modern additives (as whiteners or brighteners). The fainter emission from the *folio* is broadly distributed from 400 nm to 600 nm with a main peak at 480 nm and a shoulder at 530 nm. No emission is detected in correspondence of the darkened regions.

A clear interpretation of the spectral profiles detected in the modern passepartout, and the folio is not straightforward due to the presence of multiple fluorophores that contributed to the overall collected spectra. Nonetheless, some speculations can be done considering the typical material composition of modern and ancient paper.

The principal component of paper is cellulose, a natural linear polymer of glucose monomers linked by glycosidic C–O–C bonds. In addition to cellulose fibers, the paper contains hemicellulose, lignin, and variable amounts of fillers and whitening agents used for bleaching and strengthening the produced paper [S1]. Besides material composition, the optical and emission properties of paper are also affected by its aging. We recall here that paper aging is linked to cellulose degradation, which occurs via two main processes: hydrolysis of the C-O-C linkages between glucose monomers, and oxidation [S2].

On the basis of these considerations, it is reasonable to speculate that the emission from the passepartout (brightly intense and peaked at 430 nm) is related to the presence of brighteners, fillers, and additives typically present in modern papers. Instead, the emission from the *folio* (much lower in intensity than the emission from the *passepartout* and peaked at 480 nm with a shoulder at 530 nm) is linked to the optical emission of cellulose. Nonetheless, considering the variation of the emission properties of cellulose depending on its nature and degradation status [S3,S4], it is hard to provide its clear identification with the aid of photoluminescence spectroscopy only.


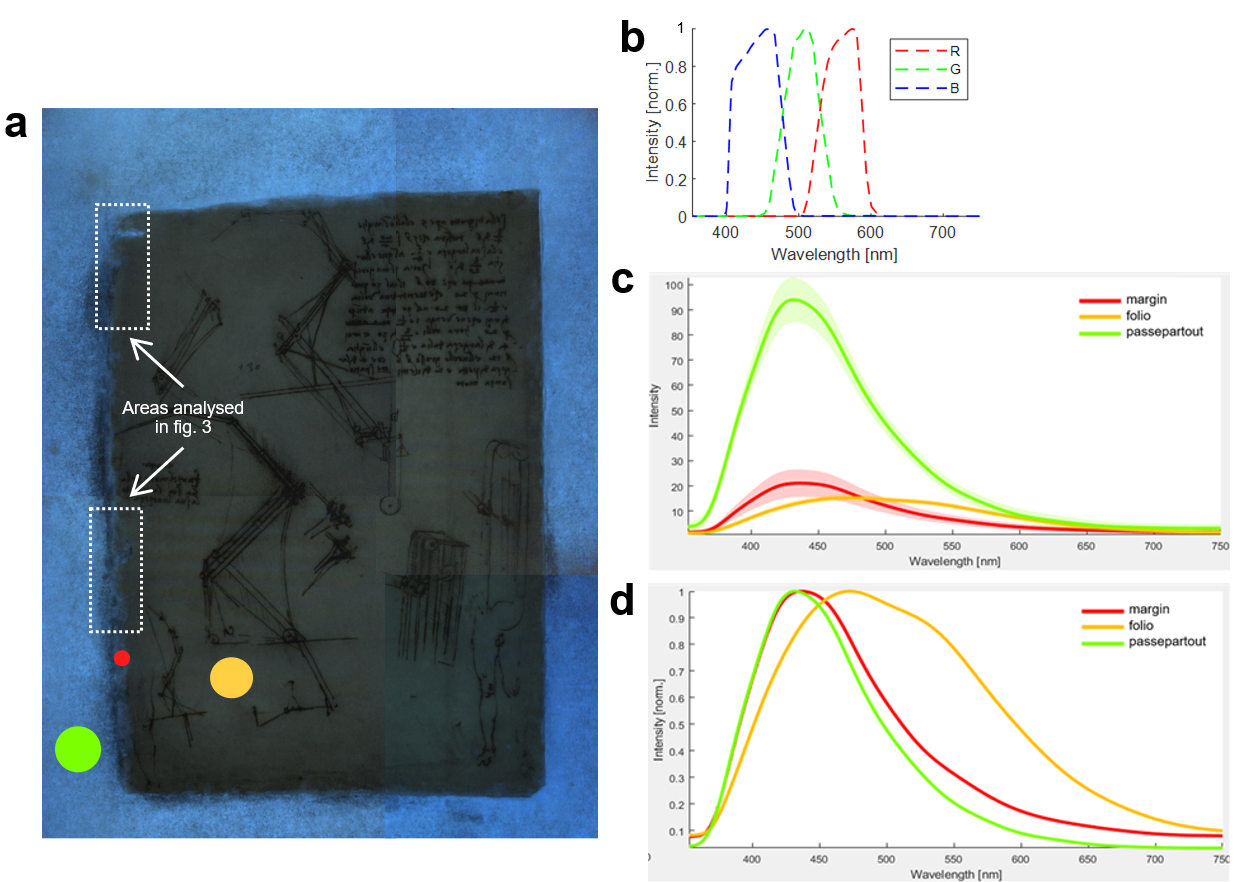


**Supplementary Fig. S3.** (**a**) False color image of the *Folio 843* reconstructed by combining the optical emission in the 400-500 nm, 480-580 nm, 500-600 nm bands as blue, green and red channels, respectively (and shown in (**b**)). The *passepartout* appears homogeneous and with an emission peak different from the one of Leonardo’s *folio*. Spectra non-normalized (**c**) and normalized (**d**) in three regions of *Folio 843*: on the *folio* (yellow), on the *passepartout* far from the edge (green) and on the edge between the two (red). Margins and *passepartout* have a similar emissions.

# Energy Dispersive X-Ray


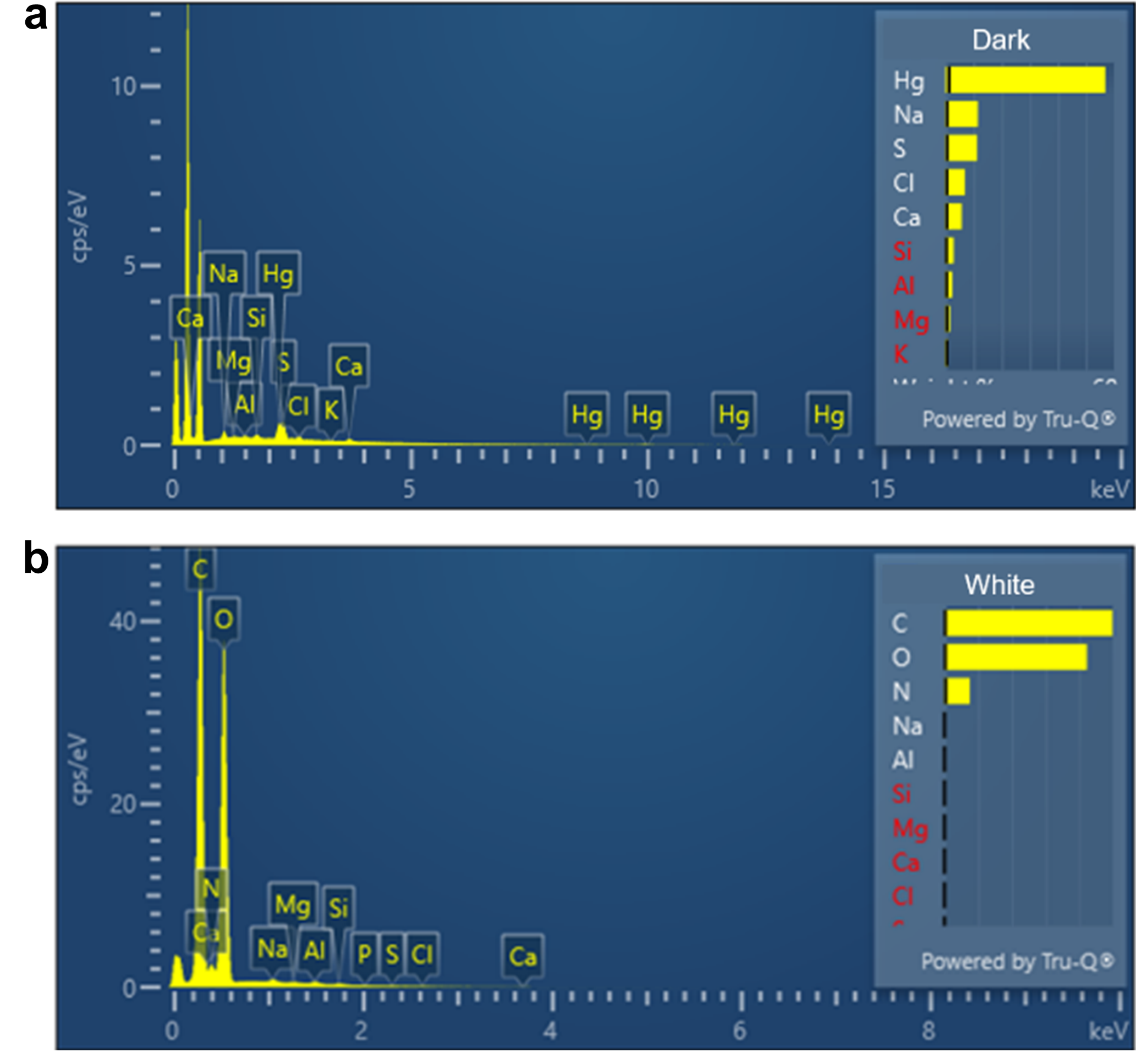


**Supplementary Fig. S4.** EDX spectra from region dark (**a**) and white (**b**). In the region dark, C and O peaks are not assigned. Instead, in region dark, there are some peaks that are not present in white region , which has been assigned to mercury and sulfur.

# μRaman spectroscopy

The system is based on a solid-state laser emitting at 785-nm in CW mode and on a spectrometer coupled to a cooled Si-based CCD camera. The detection of Raman peaks is made in the spectral range between 130 and 3000 cm^−1^ with a spectral resolution close to 10 cm^−1^. The excitation and detection units are connected to a micro-probe that allows the detection of Raman spectra at high signal-to-noise-ratio on selected spots of 15 μm in diameter at a working distance of around 2 mm [S5].

# µFTIR peaks assignment

**Supplementary table S1.** FTIR peaks assignments of the different materials of the *passepartout*

| Wavenumbers cm^-1^ | Functional group vibrations | Cellulose | Starch glue | PVAc glue | Ref. |
| --- | --- | --- | --- | --- | --- |
| 900 | C-O-C ring vibration | x |  |  | [17] |
| 930 | C-O-C ring vibration |  | x |  | [17] |
| 1014 | C-O stretching |  |  | x | [20] |
| 1027 |  |  | x |  |  |
| 1040 |  | x |  |  |  |
| 1060 |  | x | x |  |  |
| 1110 |  | x |  |  |  |
| 1160 | C-O-C asymmetric stretching | x | x |  | [17,18] |
| 1240 | C-O stretching |  |  | x | [20] |
| 1335 | C-O-H bending | x |  |  | [11] |
| 1370 | C-C-H symmetric bending | x |  | x | [11,17] |
| 1429 | -CH_2_ | x |  | x | [11] |
| 1640 | OH bending | x | x |  | [18,19] |
| 1740 | C=O stretching |  |  | x | [20] |
| 2900-2930 | C-H stretching | x | x |  | [17] |
| 3000-3300 | O-H stretching | x | x |  | [17,18] |


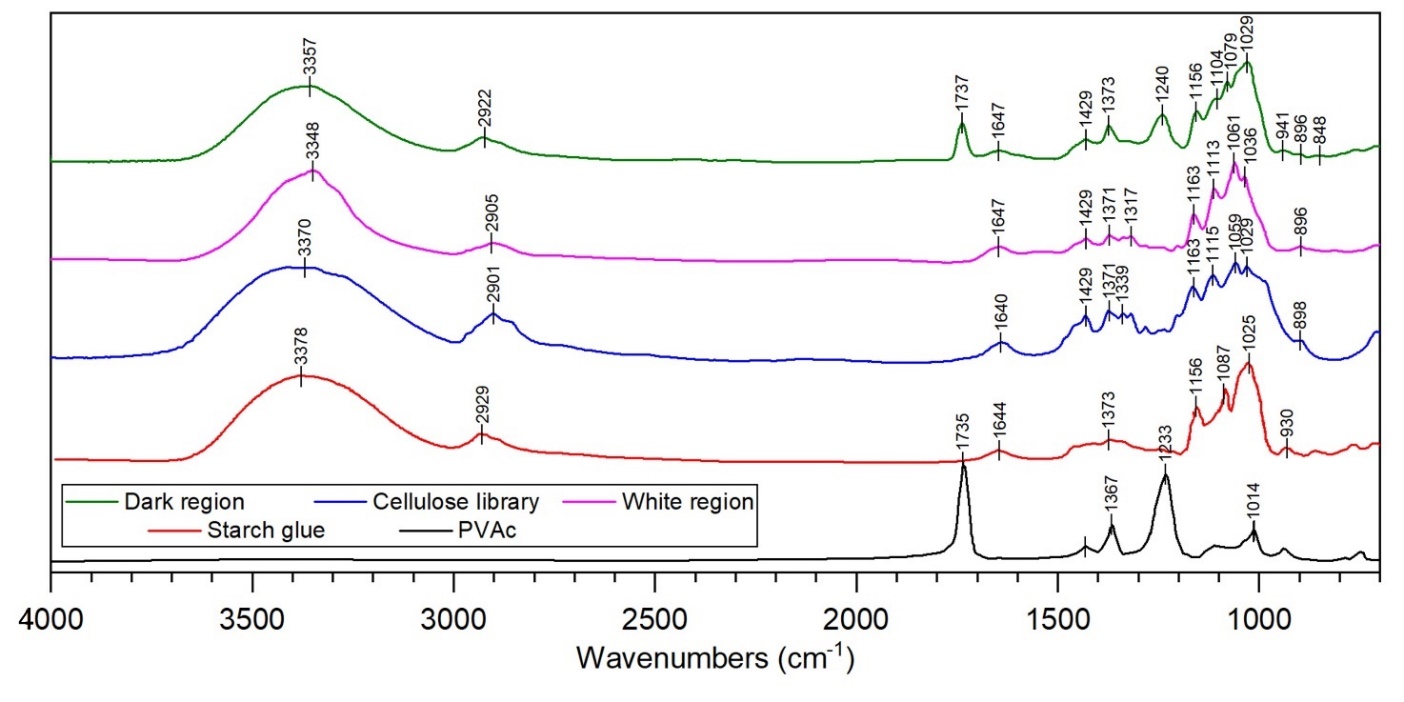


**Supplementary Fig. S5.** µATR-FTIR spectra of dark region, white region, cellulose reference, starch glue and PVAc reference samples.

# Beam damage during µXANES analyses

To assess a possible radiation damage under the micro-X-ray beam at ID21, a series of spectra were acquired on the same point, both in black and white regions. In black regions, the signal was stable, showing mainly a sulfite signal. In white region, the main peak is attributed to sulfate, but its intensity decreases under repeated XANES acquisition, and a new peak appears (2.4752 keV), which falls between the sulfide (S^-II^, 2.4737 keV) and sulfite (S^IV^, 2.4778 keV) peaks, and which could possibly be ascribed to reduced sulfur species [S6,S7] (arrow in supplementary Fig. S6a).


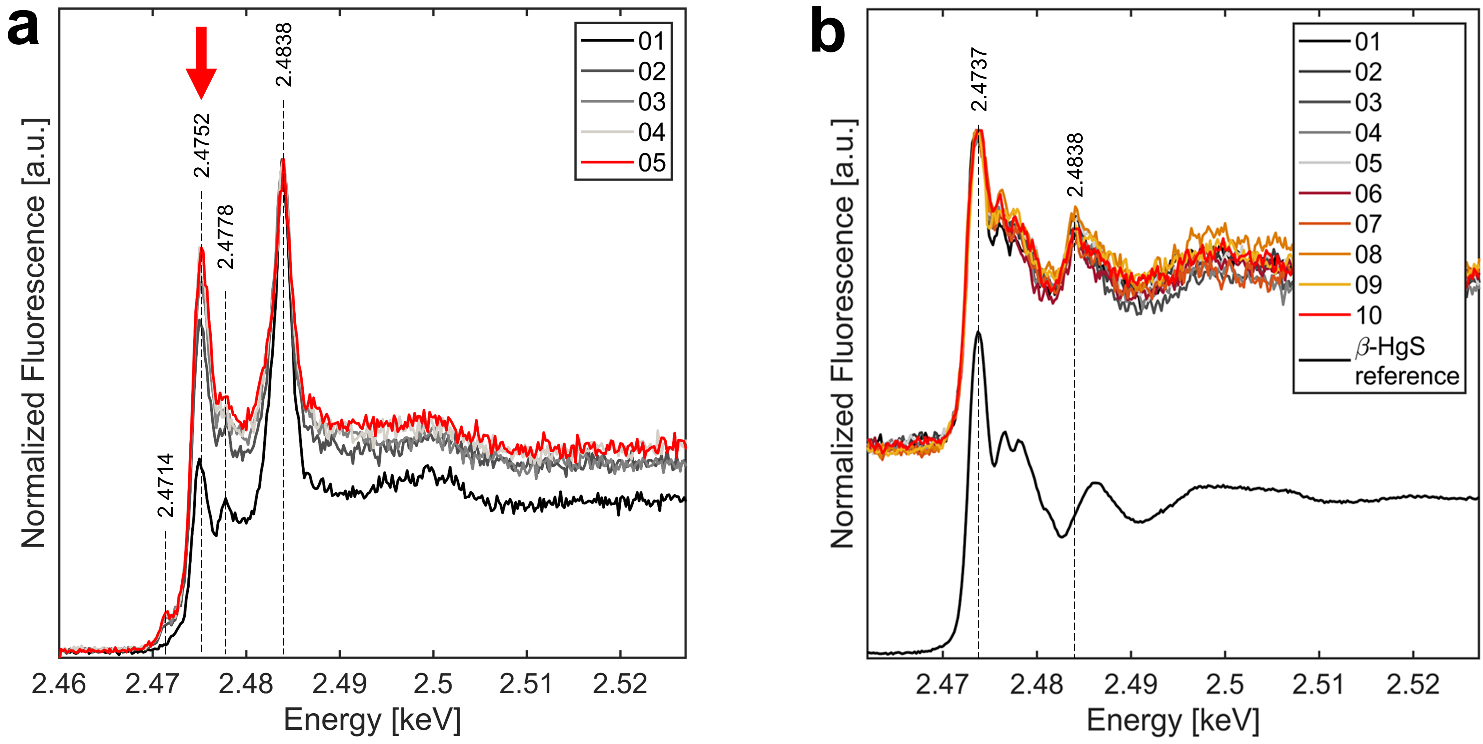


**Supplementary Fig. S6.** (**a**) Comparison of the XANES spectra of white region after several radiations of the beam. The peak at 2.4752 keV is formed by the photoreduction of sulfates. (**b**) Comparison of the XANES spectra of dark region after several radiations of the beam. No changes in the spectra are detected. The spectra are compared to the reference spectra of metacinnabar [14].

# µXRF maps


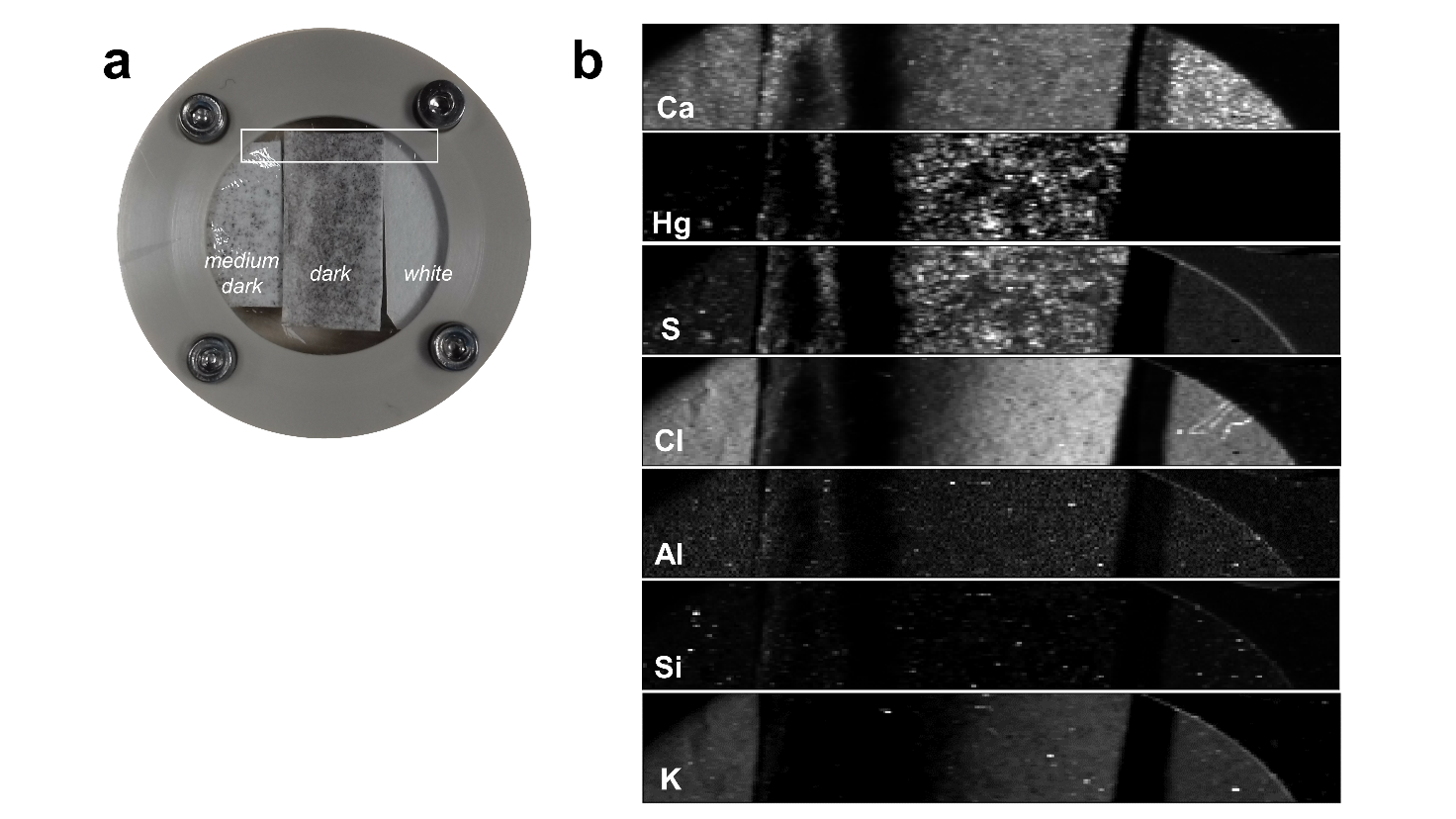


**Supplementary Fig. S7.** (**a**) Visible light image of the white, medium-dark and dark samples analyzed at ID21, mounted on the support for microscopy analysis. The white rectangle corresponds to the region where SR μXRF analysis of panels (**b**) was performed. (**b**) SR µXRF maps of Ca, Hg, S, Cl, Al, Si and K [step size (h×v), 100 × 100 μm; map size (h × v), 14 × 4 mm; exp. Time, 130 ms/pixel; energy: 4.044 keV].

# Ca and Cl K-edge macro XANES


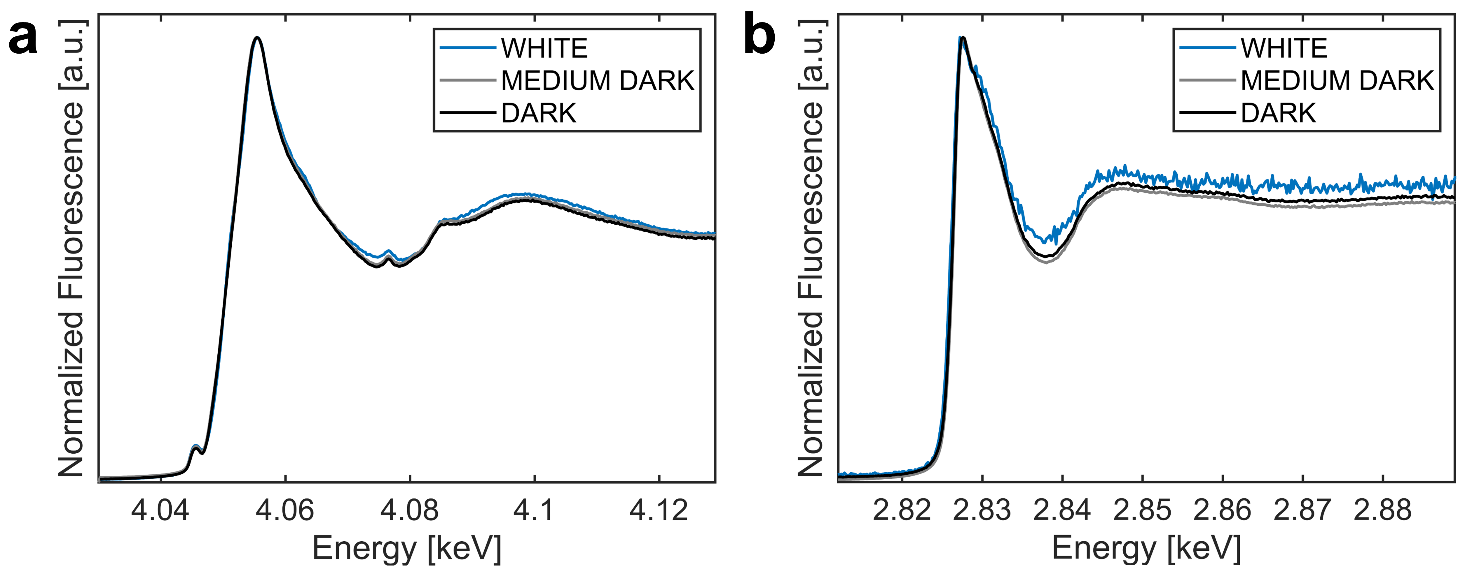


**Supplementary Fig. S8.** Series of (**a**) Ca K-edge and (**b**) Cl K-edge macro-XANES spectra recorded from white, medium-dark and dark samples.

# µXANES


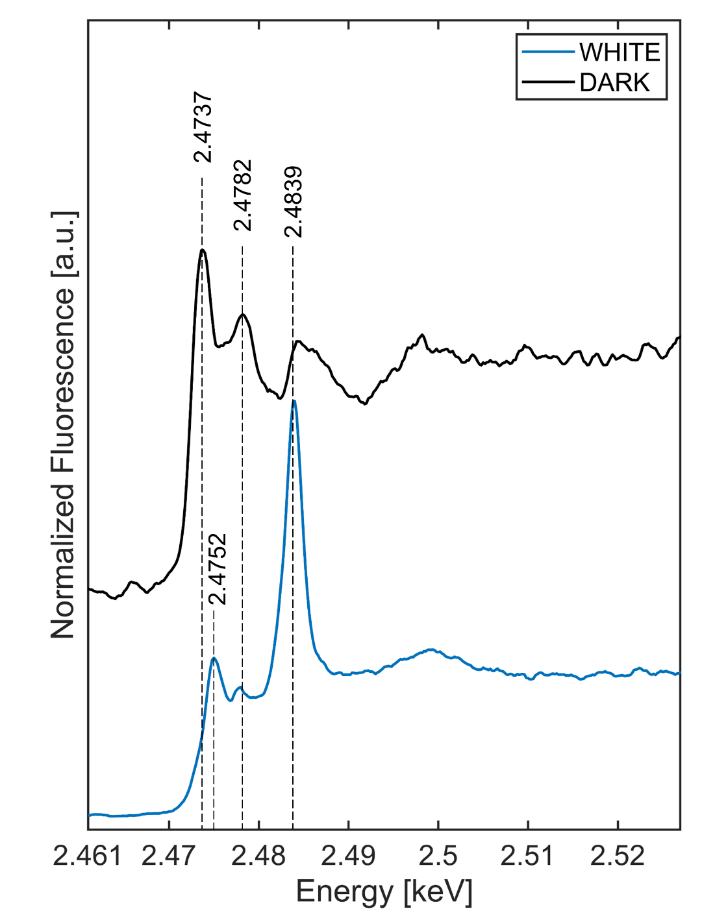


**Supplementary Fig. S9.** µXANES spectra recorded at S K-edge of white and dark regions in dark sample. Peak at 2.4737 keV corresponds to the sulfides (S^-II^), peak at 2.4752 keV possibly to sulfoxides, peak at 2.4782 keV to the sulfites (S^IV^) and peak at 2.4839 keV to the sulfates (S^VI^).

# Supplementary references

[S1] Strlic, M. & Kolar, J. *Ageing and stabilisation of paper*. (National and University library: Ljubljana, Slovenia, 2005).

[S2] Botti, S., Bonfigli, F., Nigro, V., Rufoloni, A. & Vannozzi, A. Evaluating the Conservation State of Naturally Aged Paper with Raman and Luminescence Spectral Mapping: Toward a Non-Destructive Diagnostic Protocol. *Molecules* **27**, 1712 (2022).

[S3] Łojewska, J. *et al.* Recognizing ancient papyri by a combination of spectroscopic, diffractional and chromatographic analytical tools. *Sci Rep* **7**, 46236 (2017).

[S4] Zhang, H. *et al.* Optical Characterization of Paper Aging Based on Laser-Induced Fluorescence (LIF) Spectroscopy. *Applied Spectroscopy* **72**, 913–920 (2018).

[S5] Artesani, A. et al. Combined photoluminescence and Raman microscopy for the identification of modern pigments: explanatory examples on cross-sections from Russian avant-garde paintings. Heritage Science 7, 17 (2019).

[S6] Orthous-Daunay, F.-R. et al. Speciation of sulfur in the insoluble organic matter from carbonaceous chondrites by XANES spectroscopy. Earth and Planetary Science Letters 300, 321–328 (2010).

[S7] Ganio, M., Pouyet, E. S., Webb, S. M., Patterson, C. M. S. & Walton, M. S. From lapis lazuli to ultramarine blue: investigating Cennino Cennini’s recipe using sulfur K-edge XANES. Pure and Applied Chemistry 90, 463–475 (2018)
